# Bat-Associated Pathogenic *Leptospira* spp. from Forest Fragments in Southwestern Brazilian Amazonia

**DOI:** 10.1155/2024/6633866

**Published:** 2024-01-03

**Authors:** Rair S. Verde, Maria Isabel Nogueira Di Azevedo, Daniela Dias, Tatiana Pádua Tavares de Freitas, Filipe Anibal Carvalho-Costa, Cibele Bonvicino, Walter Lilenbaum, Paulo Sérgio D'Andrea, Luciana S. Medeiros

**Affiliations:** ^1^Postgraduate Program in Health and Sustainable Animal Production in the Amazon, Federal University of Acre, Acre, Brazil; ^2^Laboratory of Veterinary Bacteriology, Biomedical Institute, Federal Fluminense University, Niteroi, Brazil; ^3^Laboratory of Biology and Parasitology of Reservoir Mammals, Oswaldo Cruz Institute, Oswaldo Cruz Foundation, Rio de Janeiro, Brazil; ^4^Laboratory of Epidemiology and Molecular Systematics, Oswaldo Cruz Institute, Rio de Janeiro, Brazil; ^5^Department of Veterinary Collective Health and Public Health, Federal Fluminense University, Niteroi, Brazil

## Abstract

Bats are known as potential carriers of different pathogens; these animals have been identified worldwide as an important reservoir of different species of *Leptospira*. Therefore, there has been an increasing interest in studying leptospirosis in neotropical bats in the Amazon. This region is a fertile ground for zoonotic diseases, given the intense process of deforestation, urbanization, opening of new agricultural frontiers, predatory hunting, effects of climate change, and loss of biodiversity. Based on this, the aim of the present study was to investigate the frequency of infection associated with the genetic characterization of pathogenic *Leptospira* spp. in bats inhabiting diverse landscapes in the southwestern region of the Amazon. To conduct the study, mist nets were installed to capture bats. Kidney samples were submitted to *Lip*L32-polymerase chain reaction and *sec*Y gene sequencing. Our results showed that 21% of neotropical evaluated bats in Southwestern Amazon were infected with *Leptospira* spp. Positive animals were found in rural, urban, periurban, and control areas. Sanguinivores had the highest frequency of positives, followed by insectivores and frugivorous bats. The species of *L. interrogans* and a potential new *Leptospira* species were identified. The frequency of *Leptospira* in bats was not influenced by landscape type, suggesting these pathogens may not be affected by landscape changes. The findings suggest that bats may serve as potential reservoirs of *Leptospira* in diverse landscapes. The presence of *Leptospira* in bats appears to be independent of the type of land use, implying that these pathogens may not be affected by small-scale changes in the environment.

## 1. Introduction

Bats are known to harbor a huge number of emerging zoonotic diseases, which can have serious implications for both human and animal health [1]. Their role in the epidemiology of diseases is even more important, as bats are susceptible to different microorganisms, including viruses, bacteria, fungi, and parasites [1–3]. From these, the bacterium *Leptospira* has been increasingly investigated over the last 2 decades [4]. Although rodents are the most important and studied reservoirs [5]; in recent years, there has been an increasing interest in studying leptospires in neotropical bats in the Amazon region, as these animals have been identified as potential carriers of different pathogens [6].

Leptospirosis is a zoonosis frequently reported worldwide and a potentially fatal disease in both humans and animals [7]. Recently, bats have been identified worldwide as an important reservoir of different species of *Leptospira* (*L. interrogans*, *L. borgpetersenii*, *L. kirschneri*, *L. fainei*, and *L. noguchii*) [6]. Currently, more than 107 species of bats infected with *Leptospira* have been reported on different continents [6]. In Brazil, several studies have reported the presence of bats infected with *Leptospira* [8–11], but few performed molecular diagnosis and genetic characterization based on DNA sequencing.

The Amazon region is a fertile ground for emergence of zoonotic diseases, given the current intense process of disorderly occupation, which harbors several extrinsic factors such as increasing deforestation, urbanization, opening of new agricultural frontiers, predatory hunting, effects of climate change, and loss of biodiversity [12]. Deforestation can increase the likelihood of contact between humans and wildlife and transmission of diseases, while fragmentation can disrupt ecosystems and alter patterns of disease transmission. A higher number of bats have been observed in urbanized areas, which is related to anthropogenic changes in the environment, with the emergence of new habitats, abundant food, and absence of bat predators [13, 14]. Those conditions facilitate the potential transmission of zoonotic pathogens by these mammals.

Therefore, the aim of the present study was to determine the frequency of infection by pathogenic *Leptospira* spp. in bats that inhabit urban, periurban, and rural fragments in the southwestern region of the Amazonia, as well as genetically characterize them to obtain molecular epidemiological inferences.

## 2. Materials and Methods

### 2.1. Study Area

**Figure 1:**
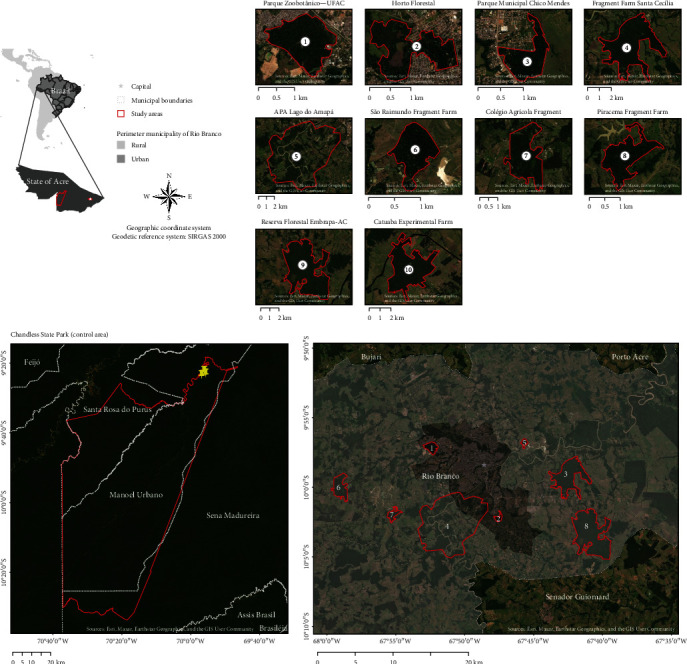
Study areas urban: 1—Parque Zoobotânico (152 ha) and 2—Parque Municipal Chico Mendes (52 ha); periurban: 3—Fragment Santa Cicília (700 ha), 4—APA Lago do Amapá (5,208 ha), and 5—São Raimundo Fragment Farm (50 ha); rural: 6—Piracema Fragment Farm (56 ha), 7—Colégio Agrícola Frangment (350 ha), and 8—Reserve Forestry Embrapa-AC (1,350 ha); and control area: Chandless State Park.

The study was conducted in the southwest region of the Brazilian Amazonia, east of the state of Acre, in nine areas with different landscapes (urban, periurban, rural, and a pristine control area). Of these, eight were in forest fragments and one was in the control area, the Chandless State Park (PEC), with an area of 695,303 ha. PEC was considered a control area as it has no habitat fragmentation impacts, environmental degradation, and is distant from urban areas (Figure 1). The east of the Acre state is dominated by induced pastures for cattle production, where forest cover (primary forest and secondary vegetation) is limited to numerous small patches. In contrast, there are areas with a landscape matrix dominated by continuous forest vegetation with deforested patches intended for seasonal agriculture. The eight selected areas were forest fragments surrounded by a mix of matrix with the presence of pastures, buildings, secondary forests, and patches of primary forests, with the control area corresponding to a continuous pristine forest. In each of the fragments, two sampling points were established, and for the control area, three.

### 2.2. Bat Collection

Collections took place between 2019 and 2021. Two nights of collection were carried out at each sampling point. We carried out a sampling effort of four collection nights in each area (32 sampling nights). In the control area, six nights were sampled, totaling a total sampling effort of 38 collection nights. Each night, eight mist nets (12 × 3 m, 19 mm mesh, Ecotone®) were installed at ground level. Captures began at sunset and ended 6 hr after net opening, with inspections every 15 min. Bat collection activities were carried out with approval from the Ethics Committee on Animal Use of the Federal University of Acre (CEUA/UFAC under no. 28/2019) and under license granted by the competent environmental agency (SISBio under no. 71451). Captured bats were placed in cotton bags for weighing and measurement. Bats were initially identified in the field with the use of field guides and identification keys available in the scientific literature [15–17]. Pregnant and/or lactating females were removed from the net and released after field identification. The others were transported to the field laboratory for biological material collection procedures. In the field laboratory, established exclusively for this purpose under Biosafety Level 3 (BSL-3) standards, bats were anesthetized and euthanized (9 : 1, 10% ketamine hydrochloride and 2% acepromazine) [18] in order to collect blood, kidneys, liver, and muscle samples for diagnosis and molecular analysis. Euthanized bats were prepared and deposited as voucher specimens in the Biology and Parasitology Laboratory of Wildlife Reservoir Mammals at IOC/Fiocruz. Bat specimens of problematic taxonomic groups were definitively identified to the species level, using integrative taxonomy approach, considering (i) morphological and morphometric analyses of the vouchers based on characters available in the systematic reviews, keys, and descriptions for each group and (ii) DNA sequencing and phylogenetic analyses.

### 2.3. Molecular Diagnosis and Genotyping *Leptospira* spp.

DNA extraction, polymerase chain reaction (PCR), DNA sequencing, and phylogenetic analysis were conducted, as previously described in D'Azeredo Torres et al.'s [19] study. Briefly, kidney samples were obtained during the necropsy and stored in sterile 2.0 mL microtubes at −20°C until molecular analysis. Detection of pathogenic *Leptospira* spp. was performed through *Lip*L32-PCR. Positive samples were subsequently submitted to a secY-nested PCR followed by DNA sequencing (410 bp) for genotyping purposes [20]. Sequences were aligned together with GenBank *Leptospira* spp. sequences from hosts of the Amazon region. Genetic distances were calculated, and a maximum likelihood phylogenetic tree was constructed using the Tamura–Nei model (TN92) in MEGA X software [21] for visual evaluation of species identification and epidemiological inferences. Regarding genetic analysis, 21 DNA sequences were obtained. Eight sequences were previously deposited on GenBank in our first study under accession numbers OQ793707–OQ793714 [22]. The remaining sequences (*n* = 13) were deposited herein under accession numbers OQ992783–OQ992795.

### 2.4. Statistics

In order to evaluate an association between the positivity frequency for pathogenic *Leptospira* spp. and the different types of landscapes, we utilized Pearson's *χ*^2^-test. We verified the normality of the data through the Shapiro–Wilk test. A value of *p* < 0.05 was considered statistically significant. All statistical analyses were conducted using the “vegan” packages [23] (R Core Team, 2022) in R software version 3.0.3.

## 3. Results

A total of 209 bats belonging to two families (Phyllostomidae and Vespertilionidae), seven subfamilies, 20 genera, and 33 species (Table 1) were captured in the nine studied areas with different landscapes. Of these, 45 bats were from urban areas, 51 from periurban areas, 69 from rural areas, and 44 from control areas.

**Figure 2:**
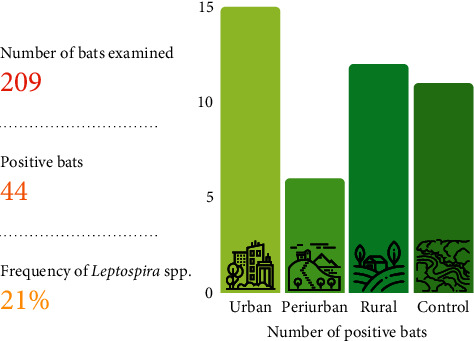
Number of positive cases of infection by *Leptospira* spp. in bats according to landscape types (control area = continuous forest).

The *LipL32*-PCR revealed that 44 bats (21%) were positive for pathogenic *Leptospira* spp. The positive hosts belonged to 16 different species, including *Artibeus planirostris* (*n* = 10), *Artibeus lituratus* (*n* = 6), *Carollia perspicillata* (*n* = 4), and *Desmodus rotundus* (*n* = 4) (*Supplementary 1*). We have identified, for the first time, the species *Choeroniscus minor*, *Lophostoma brasiliense*, and *Rhinophylla fischerae* as carriers of *Leptospira* spp. (*Supplementary 1* and *Supplementary 2*). Regarding landscape origin, from the positive bats, 15 (7.2%) were from rural areas, 12 (5.7%) from urban areas, 11 (5.3%) from control areas, while only six bats (2.9%) from periurban areas. The difference in frequency between type of landscapes was not statistically significant (*X*^2^ = 3.9325, df = 3, *p* > 0.05) (Figure 2).

**Figure 3:**
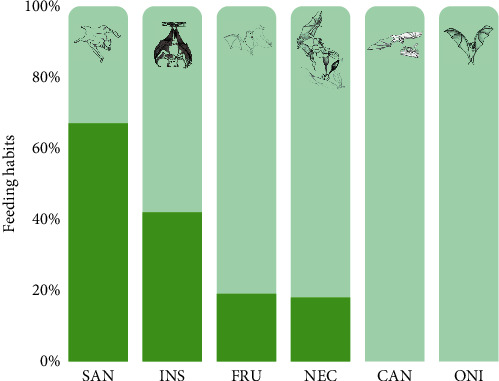
Frequency of positive cases of infection by *Leptospira* spp. according to bat feeding habits (CAN, carnivore; FRU, frugivore; INS, insectivore; NEC, nectarivore; ONI, omnivore; and SAN, sanguinivore). Drawings by Izailene Saar.

The bat species have different feeding habits, such as frugivorous (80.4%), insectivores (9.1%), nectarivores (5.3%), omnivores (2.9%), sanguinivores (1.4%), and carnivores (0.9%) (Table 1). The sanguinivores had a higher frequency of positives at 67% (*n* = 3), followed by insectivores at 42% (*n* = 19), and frugivorous at 19% (*n* = 32) (Figure 3). When we tested the frequency of positives against the type of feeding habit, a significance was obtained (*X^2^* = 14.831, *df* = 6, *p* < 0.05).

Pairwise/Blast/NCBI comparisons with the GenBank *sec*Y gene dataset obtained here revealed 12 sequences with >99% of identity with *L. interrogans* and nine sequences with no identity (<90%) with previously deposited *Leptospira* species. Due to the possible identification of a new species, a previous study was conducted to deeper investigate and genetically characterize these unknown leptospires [22].

**Figure 4:**
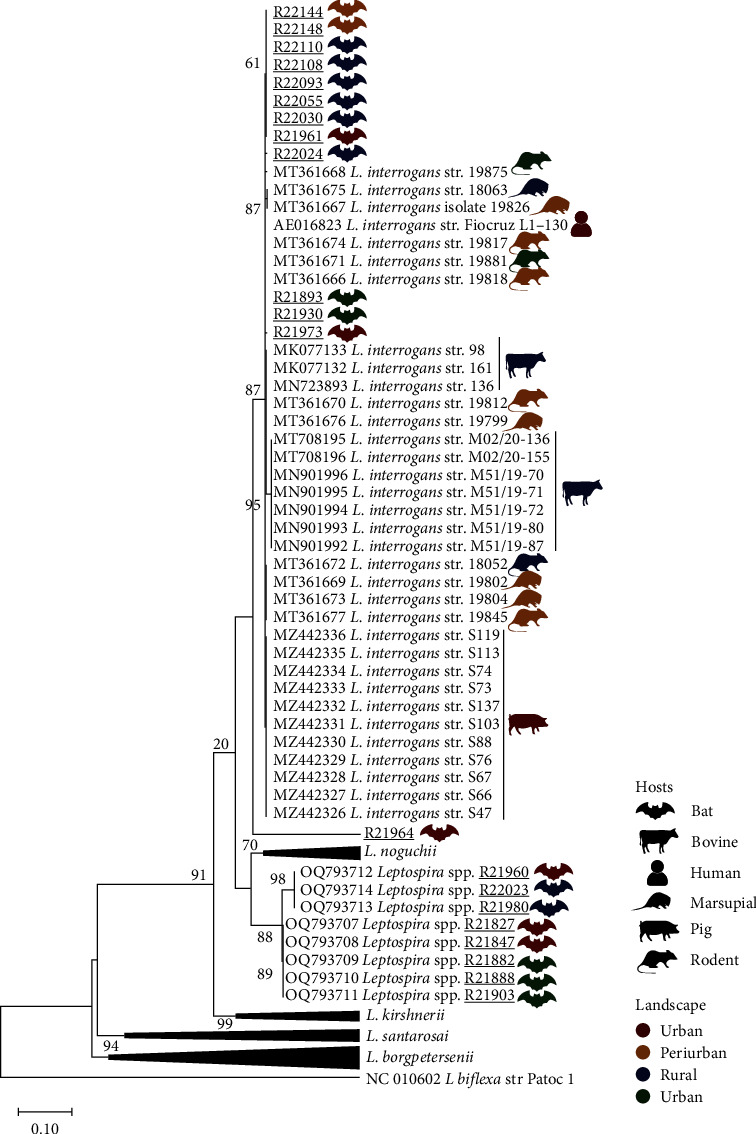
Maximum-likelihood phylogenetic tree inferred from *sec*Y gene sequences, including the main pathogenic *Leptospira* species and sequences from this study (underlined). Vectors represent hosts and colors the landscape type, as shown in the figure. Numbers at nodes are bootstrap values greater than 50. *Leptospira biflexa* is the outgroup taxa. Additional information, including host species and exact geographical localization of sequences used on phylogenetic analysis are shown in *Supplementary 2*.

Phylogenetic analysis based on ML-TN92 (Figure 4) confirmed species identification of 12 *L. interrogans* species and reaffirmed the topology of our previous study that showed a distinct clade from the new *Leptospira* spp. species circulating in bats from Amazon region. Moreover, one sequence (R21964) did not cluster with any previously known *Leptospira* species and neither to the new species detected. Additionally, a high genetic distance was found between deposited sequences (0.07–0.18, i.e, only 93%–82% of identity) and can be another potential new undescribed *Leptospira* species.

The only host-specific cluster observed was the one including sequences belonging to *Leptospira* spp. from bats as previously evidenced by Di Azevedo et al. [22] (Figure 4). Regarding the remaining *Leptospira* sequences from Amazon region, no specific clusters were observed according to host or landscape type (Figure 4, *Supplementary 2*). Importantly, *L. interrogans* sequences from the present study clustered together with sequences from a great variety of hosts, including the strain Fiocruz L1-130 from human origin.

## 4. Discussion

The findings suggest that bats may serve as potential reservoirs of *Leptospira* in diverse landscapes. The presence of Leptospira in bats appears to be independent of the type of land use, implying that the presence of these pathogens in bats may be affected by small-scale changes in the environment mainly considering the presence of other hosts, the presence of wastewater, and bodies of water in these areas. Although urban forest fragments have a high prevalence of *Leptospira*, the same pattern is observed in rural forest fragments and continuous forests (control area). The results may be attributed to certain biological traits of bats, such as the formation of large colonies, long-distance migration [24], extended lifespan [25], and high adaptability of many species [2]. Urban fragments provide suitable vegetation structures for roosting and feeding, which allows bat species to persist [13] and demonstrates their tolerance to landscape alterations (such as, forest fragmentation) [2]. The formation of large colonies enables transmission of the disease between bats, while their ability to fly and migrate over great distances could connect urban, rural, and wild cycles of leptospirosis [26]. Additionally, the longevity of bats could facilitate the spread of the bacteria through urine over extended periods in various environments and animals.

Regarding feeding habits, our observations indicate that *Leptospira* spp. can infect bats regardless of their feeding habits or population density. However, no cases of infection were detected in carnivores and omnivores. In general, bats, which have behavioral habits of forming colonies, the habit of licking each other in order to cleaning up, and females often regurgitate to feed their offspring. These behaviors promote close proximity among animals, consequently increasing the likelihood of transmitting pathogens. Furthermore, there is a greater risk of exposure for frugivorous bats, which might share their food with rodents [27]. Additionally, species like *Desmodus rotundus*, with their excellent abilities to move by walking or jumping on the ground [28], can share a direct environment, increasing the risk of contamination compared to frugivores, carnivores, and omnivores. Other factors such as the type of shelter, shelter sharing, and urbanization may contribute to bat/*Leptospira* interactions. Urban afforestation and public lighting attract insects, becoming potential sources of food and shelter for both insectivorous and frugivorous bats, predisposing them to co-occur. The concentration of urine in a particular shelter and environment increases with the number of bats and the size of that refuge. Therefore, *Leptospira* spp. can remain present for longer periods, potentially increasing the risk of infection.

Interestingly, the frequency of infection varies among bat species, even among congeners, indicating that some species may be better adapted to carrying leptospires than others. *Artibeus planirostris*, *Artibeus lituratus*, and *Carollia perspicillata* had at least five infected individuals. Matthias et al. [29] found a similar pattern in a study conducted in Peru. Notably, infected bats were also found in species with high, medium, and low abundance, indicating that infection is independent of the local abundance of bat populations, a result that was also reported in a study in Colombia [26].

Based on the phylogenetic analysis of *SecY* sequences, the bats in the study maintain a genetically diverse group of leptospires. It is not surprising, considering the number of different bat species in the region. However, the detection of a new pathogenic species of *Leptospira* circulating in different environments deserves attention [22]. This fact becomes even more concerning due to the intense deforestation processes that the Brazilian Amazon has been suffering, generating environmental changes, and ecological disturbances that may facilitate contact between humans and wild species of vectors and reservoirs. This fact could contribute to the emergence of zoonotic diseases [12]. It is important to highlight that bats are important hosts of reservoirs and disseminators of multiple pathogenic *Leptospira* species, and as flying mammals, they can reach long distances, including urban areas [26].

The emergence of the zoonotic diseases, such as the COVID-19 pandemic, with bats being pointed as potential responsible for its origin, highlights the need to increase knowledge about the zoonotic pathogens and implement rapid and aggressive responses to track spillover events in order to avoid the repetition of situations like the one recently experienced. This study was a starting point to better delineate the ecological aspects of *Leptospira* spp. infection on bats considering the animal and bacteria species on diverse Amazonian landscapes. The homogeneity of strains, independent of the type of land use, suggests that the bat's ability to permeate habitats is a key factor in the distribution of *Leptospira* spp. and haplotypes. However, additional studies are needed to better understand the role of these flying mammals in the maintenance and transmission of *Leptospira* spp. and to clarify their relationship with the abiotic and biotic environment to determine the mechanisms of transmission and persistence of the pathogens in an ecological context.

## Figures and Tables

**Table 1:** Bat species, with information on the number of positive and negative cases of *Leptospira* spp., the frequency of positive cases, and feeding habits.

Bat species	Negative	Positive	Number of captures	Frequency of cases	Feeding habits
Family phyllostomidae Subfamily carollinae					
* Carollia brevicauda* (Schinz, 1821)	15	3	17	0.18	Frugivore
* Carollia perspicillata* (Linnaeus, 1758)	28	5	33	0.15	Frugivore
Subfamily desmodontinae					
* Desmodus rotundus* (E. Geoffroy, 1810)	1	2	3	0.67	Sanguinovore
Subfamília glossophaginae					
* Anoura caudifer* (E. Geoffroy, 1818)	1	0	1		Nectarivore
* Choeroniscus minor* (Peters, 1868)	0	1	1	1.00	Nectarivore
* Glossophaga soricina* (Pallas, 1766)	4	1	5	0.20	Nectarivore
Subfamília lonchophyllinae					
* Hsunycteris pattoni* (Woodman & Timm, 2006)	4	0	4		Nectarivore
Subfamilia micronycterinae					
* Micronycteris microtis* (Miller, 1898)	1	0	0		Insetivore
Subfamilia phyllostominae					
* Gardnerycteris crenulatum* (E. Geoffroy, 1803)	3	2	5	0.40	Insetivore
* Lophostoma brasiliense* (Peters, 1867)	0	2	2	1.00	Insetivore
* Lophostoma silvicolum* (d'Orbigny, 1836)	2	2	4	0.50	Insetivore
* Phylloderma stenops* (Peters, 1865)	1	0	0		Onivore
* Phyllostomus elongatus* (E. Geoffroy, 1810)	4	0	4		Onivore
* Phyllostomus hastatus* (Pallas, 1767)	2	0	2		Onivore
* Tonatia maresi Williams* (Willig & Reid, 1995)	1	1	2	0.50	Insetivore
* Trachops cirrhosus* (Spix, 1823)	1	0	1		Carnivore
* Vampyrum* spectrum (Linnaeus, 1758)	1	0	1		Carnivore
Subfamilia rhinophyllinae					
* Rhinophylla fischerae* (Carter 1966)	0	1	1	1.00	Frugivore
* Rhinophylla pumilio* (Peters, 1865)	1	0	1		Frugivore
Subfamilia stenodermatinae					
* Artibeus anderseni* (Osgood, 1916)	1	0	0		Frugivore
* Artibeus concolor* (Peters, 1865)	1	0	0		Frugivore
* Artibeus lituratus* (Olfers, 1818)	19	6	25	0.24	Frugivore
* Artibeus obscurus* (Schinz, 1821)	5	2	7	0.29	Frugivore
* Artibeus planirostris* (Spix, 1823)	41	10	51	0.20	Frugivore
* Mesophylla macconnelli* (Thomas, 1901)	0	0	0		Frugivore
* Platyrrhinus incarum* (Thomas, 1912)	2	0	2		Frugivore
* Platyrrhinus brachycephalus* (Rouk & Carter, 1972)	1	0	1		Frugivore
* Sphaeronycteris toxophyllum* (Peters, 1882)	1	0	1		Frugivore
* Sturnira giannae* (Velazco & Patterson, 2019)	6	0	6		Frugivore
* Sturnira tildae* (de la Torre, 1959)	1	1	2	0.50	Frugivore
* Uroderma bilobatum* (Peters, 1866)	12	4	16	0.25	Frugivore
* Uroderma magnirostrum* (Davis, 1968)	4	0	4		Frugivore
Família vespertilionidae					
* Myotis riparius* (Handley, 1960)	6	1	6	0.16	Insetivore
Total	170	44	209	0.46	

## Data Availability

The data supporting the results and conclusions presented in this manuscript are available for public access, promoting transparency, and replicability of the research. All information related to the molecular analyses and sequencing used in the study has been deposited in Genbank, a database of genetic sequences maintained by the National Center for Biotechnology Information (NCBI), which can be accessed through the following link: https://www.ncbi.nlm.nih.gov/genbank/. To facilitate direct access to the specific samples used in this study, we have provided the accession numbers of each sample in the supplementary material attached to this manuscript. With this information, readers can obtain the relevant data through Genbank and examine the genetic sequences or any other relevant details for the replication and analysis of the presented results. We emphasize the importance of open sharing of scientific data as an essential means for validation and furthering research. By making our data available in Genbank and providing the accession numbers in the supplementary material, we hope that researchers and interested individuals can explore and build upon the findings of this study in an ethical and collaborative manner. We appreciate the interest in our research and encourage anyone to use the available data to advance scientific knowledge and contribute to progress in their respective fields of expertise.

## Supplementary Materials

Supplementary 1 There are some pictures of the bat species captured during the study.

Supplementary 2 Additional information, including host species and exact geographical location of sequences used on phylogenetic analysis and GenBank accession numbers of samples.
