# Supplementary figures and images for: Bat-Associated Pathogenic Leptospira spp. from Forest Fragments in Southwestern Brazilian Amazonia
Source: Transbound Emerg Dis. 2024 Jan 3;2024:6633866. doi: 10.1155/2024/6633866 (PMC12017250; doi:10.1155/2024/6633866)

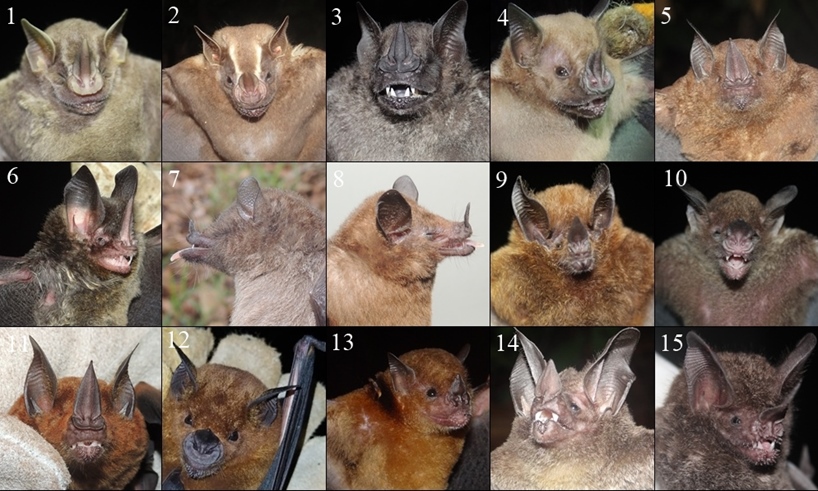

Supplement: Supplementary 2 — Additional information, including host species and exact geographical location of sequences used on phylogenetic analysis and GenBank accession numbers of samples. [file 6633866.f2.jpg]
